# Latin America and the Caribbean: Challenges and Opportunities for HIV Treatment and Prevention

**DOI:** 10.1002/jia2.70170

**Published:** 2026-07-25

**Authors:** Mateo Prochazka, Kelika A. Konda, Omar Sued, Hortencia Peralta, Andrea Boccardi, Thiago S. Torres, Monica Alonso, Valdilea Veloso, Beatriz Grinsztejn

**Affiliations:** ^1^ World Health Organization Geneva Switzerland; ^2^ Keck School of Medicine University of Southern California Los Angeles California USA; ^3^ Universidad Peruana Cayetano Heredia Lima Peru; ^4^ Pan American Health Organization Washington DC USA; ^5^ UNAIDS Brasilia Brazil; ^6^ Fundação Oswaldo Cruz Instituto Nacional de Infectologia Evandro Chagas Rio de Janeiro Brazil

**Keywords:** Caribbean, HIV care, HIV cascade, HIV prevention, Latin America, PrEP

## Abstract

**Introduction:**

In Latin America, there are rising numbers of new HIV acquisitions, while the Caribbean has achieved decreases in both new acquisitions and AIDS‐related deaths. The Latin American and Caribbean (LAC) region has worked towards widely available treatment for people with HIV (PWHIV) within public health systems and, in Latin America, has relied principally on domestic financing to do so.

**Discussion:**

While new methods of prevention are in the process of being incorporated into regional HIV programmes, the HIV cascade of care remains below the UNAIDS goals. Only 17 of the 32 LAC countries (53%) report on the three pillars. Only Chile achieved >95% in two of the three pillars, with 95% of PWHIV knowing their status and 95% of people on treatment having a suppressed viral load. However, only 75% of those who know their status were on treatment. In the Caribbean, very little data is available, with all three cascade pillars missing for six of the countries. Only the Bahamas reached the 95% threshold for any of the cascade steps. Additionally, in the Caribbean, the viral suppression step of the cascade is much lower on average than in Latin America, with no countries reaching 80%. Biomedical HIV prevention with pre‐exposure prophylaxis (PrEP) and post‐exposure prophylaxis (PEP) is still being adopted and scaled up among populations in need. Scale‐up of prevention efforts remains hindered by structural limitations of the health system, legal and policy limitations such as continuing to require informed consent for HIV testing and policies that block task‐shifting, as well as persistent stigma. Recent reductions in international funding for HIV may adversely influence HIV programmes in LAC, which have relied on these for initial scale‐up of innovations.

**Conclusions:**

Accelerating differentiated and simplified delivery of PrEP and PEP, alongside sustained progress towards universal treatment coverage and viral suppression, are all essential for translating existing scientific advances into measurable progress towards HIV elimination in the region.

## 1 Introduction

Global estimates show significant progress in reducing new HIV acquisitions worldwide, yet trends in Latin America follow a different trajectory, with a 13% increase in annual cases between 2010 and 2024 [1, 2]. Conversely, the Caribbean achieved sustained declines in both new cases and AIDS‐related deaths in the same period (−21% and −62%, respectively) [3]. The annual number of AIDS‐related deaths in the Latin American and Caribbean (LAC) region remains unacceptably high, with 27,000 in Latin America plus 4800 in the Caribbean in 2024. These results underscore the need to improve the HIV care and prevention continuum [4].

The development of highly effective, well‐tolerated antiretroviral treatment (ART) has facilitated single‐pill regimens, improving outcomes, reducing costs and simplifying logistics [5]. However, a comprehensive approach is needed to end the epidemic, including expanding HIV testing, providing immediate treatment and effective linkage to pre‐exposure prophylaxis (PrEP) or post‐exposure prophylaxis (PEP) [6]. PrEP products, including long‐acting formulations, have the potential to significantly reduce HIV incidence [7]. However, PrEP implementation, currently limited to oral PrEP, lacks sufficient pace in LAC [8]. Although countries in LAC have contributed importantly to HIV clinical trials and implementation science research [9, 10, 11], access to these interventions remains uneven.

In this commentary, we examine the epidemiology of HIV in LAC, evaluate emerging biomedical prevention and treatment approaches, and identify region‐specific barriers that hinder effective scale‐up. Our goal is to inform a multisectoral response that accelerates innovations and supports countries in advancing towards eliminating HIV.

### 1.1 Epidemiology of HIV in LAC

In 2024, UNAIDS estimated that 2.8 million people are living with HIV (PWHIV) in LAC, 140,000 new HIV acquisitions and 32,000 AIDS‐related deaths [2, 3, 12]. Regionally, approximately 86% of PWHIV know their status, but only 71% of them receive ART, and just 66% of those on ART achieve viral suppression [2, 3]. The overall cascade is approximately equal between men and women; however, available data are worse among children aged 0–14, with only 56% knowing their HIV status, 44% receiving ART and 39% achieving viral suppression [13].

In Latin America, most new acquisitions occur among key populations and their partners [13]. These groups primarily include men who have sex with men (MSM), transgender people, particularly *travesti* (an identity distinct from transgender woman, widely used in Latin America), sex workers, people who use drugs, some indigenous communities, people in prisons and migrants [2]. One recent study reported an annualized HIV incidence among sexual and gender minorities not using PrEP/PEP of 2.62% in Brazil (95% confidence interval [CI]: 1.78−3.43) and 6.69% (95% CI: 4.60−8.69) in Peru [14].

Caribbean countries face more generalized HIV epidemics, with an adult prevalence (15−49 years) of approximately 1.2% [3]. However, the epidemic is also mixed, with a disproportionately high burden among key populations, within whom HIV prevalence remains markedly higher than in the general population. These dual dynamics underscore the need for integrated responses addressing both broad population transmission and the specific vulnerabilities of key populations [15]. In Haiti, one of the countries with the highest HIV burden, recent estimates place adult prevalence around 2% [16].

Across the LAC region, a substantial proportion of PWHIV are diagnosed late, contributing to elevated morbidity and mortality associated with advanced HIV disease [17, 18, 19]. Structural challenges, including fragmentation of the health system, geographic barriers and disruptions in continuity of HIV services, undermine timely testing, linkage and retention in care, hampering the epidemic response [20].

Migration is also an important issue shaping the evolving epidemiology of HIV in LAC. By the end of December 2025, there were more than 7.9 million Venezuelan refugees and displaced migrants globally, with over 85% hosted by LAC countries [21]. Colombia, Peru, Ecuador and Chile have received the most migrants, including thousands of PWHIV due to medication stockouts and other health system problems in Venezuela [22]. In Colombia, which hosts the largest number of Venezuelan migrants worldwide (2.81 million, May 2025), a recent study found a 0.9% (95% CI: 0.6%−1.4%) HIV prevalence among this population, almost double the 0.5% (95% CI: 0.4%−0.6%) HIV prevalence among Colombia's adult population [23].

### 1.2 The 95‐95‐95 Targets and HIV Treatment in LAC

In LAC, 17 of the 32 countries (53%) report on the three 95‐95‐95 goals (Table 1) and only Chile achieved >95% in two of the three pillars. In the Caribbean, very little data is available, with all three cascade pillars missing for six of the countries. Only the Bahamas reached the 95% threshold at the cascade step. In the Caribbean, viral suppression is much lower than in Latin America, with no countries reaching 80% (Table 1). Country‐wide cascades mask important subnational differences. For example, several Amazonian communities in Peru and Brazil have higher HIV prevalence, aggravated by isolation, poor health infrastructure, lack of culturally adapted interventions, migration, poverty and sexual dynamics between these communities and urban centres [24, 25, 26].

**TABLE 1:** Country‐level data on HIV incidence and key indicators on treatment and prevention (UNAIDS, 2024; PAHO, 2024).

Country	People living with HIV (95% CI)	New HIV acquisitions with HIV number and 95% CI	People living with HIV who know their status number (%)	People who know their status and are on ART number (%)	People who are on ART and with viral suppression number (%)	AIDS‐related deaths number and 95% CI	People who used PrEP at least once in a year	World Bank classification
**Latin America**								
Argentina	140,000 (130,000−150,000)	4200 (3600−5200)	94	84	NA	1400 (<1000−1700)	3914	UMIC
Bolivia	31,000 (27,000−37,000)	2000 (1600−2600)	NA	NA	85	<1000 (<500−<1000)	N/A	LMIC
Brazil	1,000,000 (930,000−1,100,000)	51,000 (48,000−54,000)	89	82	96	14,000 (10,000−17,000)	165,473	UMIC
Chile	91,000 (81,000−100,000)	3400 (2700−4000)	95	75	95	N/A	3150	HIC
Colombia	230,000 (190,000−310,000)	13,000 (8000−23,000)	79	NA	NA	3700 (2100−6700)	9660	UMIC
Costa Rica	19,000 (16,000−22,000)	<1000 (<500−1200)	69	90	NA	<200 (<200−<500)	2799	UMIC
Ecuador	51,000 (44,000−58,000)	2200 (1600−3000)	92	89	92	<500 (<500−<1000)	3887	UMIC
El Salvador	23,000 (20,000−25,000)	<1000 (<1000−1100)	93	82	95	<500 (<500−<500)	5988	UMIC
Guatemala	35,000 (33,000−37,000)	1500 (1500−1800)	94	83	85	<500 (<500−<1000)	1222	UMIC
Honduras	20,000 (17,000−22,000)	<1000 (<500−<1000)	83	90	95	<500 (<500−<1000)	3918	LMIC
Mexico	380,000 (330,000−430,000)	19,000 (15,000−26,000)	80	81	93	5100 (3600−6700)	30,707	UMIC
Nicaragua	12,000 (9000 −15,000)	<1000 (<500−1100)	NA	NA	87	<200 (<100−<500)	307	LMIC
Panama	30,000 (27,000−33,000)	1500 (1300−1700)	77	NA	93	<500 (<500−<500)	740	UMIC
Paraguay	22,000 (18,000−27,000)	1400 (<1000−2100)	91	64	85	<500 (<500−<1000)	1061	UMIC
Peru	110,000 (97,000−130,000)	6300 (4800−8400)	87	92	83	<1000 (<1000−1200)	5443	UMIC
Uruguay	16,000 (12,000−22,000)	<1000 (<500−1700)	94	80	NA	<200 (<100−<500)	491	HIC
Venezuela	100,000 (91,000−110,000)	7600 (6300−9200)	79	71	NA	N/A	N/A	UMIC
**Caribbean**								
Antigua and Barbuda	800 (700−910)	40 (28−57)	NA	NA	NA	7 (5−9)	8	HIC
Bahamas	4000 (3500−4500)	53 (37−75)	95	74	68	82 (58−110)24	140	HIC
Barbados	2400 (2100−2700)	94 (64−130)	93	60	58	66 (51−80)	107	HIC
Belize	3900 (3200−3600)	190 (150−240)	77	50	38	100 (88−120)	83	UMIC
Cuba	43,000 (37,000−48,000)	1800 (1300−2200)	81	68	60	380 (190−580)	3998	UMIC
Dominica	210 (170−260)	8 (4−13)	NA	NA	NA	3(2−5)	1	UMIC
Dominican Republic	85,000 (65,000−99,000)	4000 (2300−5600)	92	66	64	1300 (770−1900)	4551	UMIC
Guyana	11,000 (7700−15,000)	560 (360−1000)	92	69	65	120 (57−300)	5237	UMIC
Haiti	150,000 (13,000−170,000)	5800 (4200−8700)	87	87	74	1300 (1000−1900)	33,375	LMIC
Jamaica	28,000 (26,000−31,000)	1100 (890−1500)	NA	54	50	NA	602	UMIC
Saint Kitts and Nevis	240 (200−270)	12 (9−16)	NA	NA	NA	3 (2−4)	NA	HIC
Saint Lucia	650 (560−770)	34 (25−45)	NA	NA	NA	25 (18−32)	5	UMIC
Saint Vincent and the Grenadines	670 (580−760)	17 (11−26)	NA	NA	NA	14 (10−18)	NA	UMIC
Suriname	7900 (7000−8800)	460 (360−580)	53	49	45	220 (170−290)	NA	UMIC
Trinidad and Tobago	NA	NA	NA	NA	NA	NA	NA	HIC

*Note*: Orange indicates ≤80%, pink 81%–90%, yellow 91%–94% and green ≥95%, the stated UNAIDS goal.

Abbreviations: ART, antiretroviral therapy; CI, confidence interval; HIC, high‐income country; LMIC, lower‐middle‐income country; NA, not available; PrEP, pre‐exposure prophylaxis; UMIC, upper‐middle‐income country.

Among low‐ and middle‐income countries, Latin America was the first region to provide ART within public health systems, with Brazil and Argentina producing and even exporting ART to neighbouring countries in the 1990s [27]. Currently, ART programmes in Latin America have the highest level of domestic HIV funding, with domestic sources covering more than 80% of the overall response and 99% of ART costs [28]. A notable treatment success is the widespread adoption of the single pill tenofovir/lamivudine/dolutegravir or lamivudine/dolutegravir as a once‐a‐day regimen.

Across LAC, diagnosis, linkage‐to‐care and disengagement are important limitations to ART coverage. Structural factors dominate barriers to testing. In almost all countries, HIV testing requires signed informed consent, which acts as a disincentive. Some country regulations impede rapid‐test use by non‐laboratory personnel, and self‐tests are rare [29, 30]. These limitations exacerbate late diagnosis (defined as CD4<200 cells/mm^3^), which remains a pressing concern at 35% [19, 31].

Mental health comorbidities, including depression and substance use disorders, represent critical but largely unaddressed barriers to sustained engagement in HIV treatment in LAC. Treatment interruptions contribute to more than 50% of advanced HIV disease, often mediated by depression, substance use including alcohol, and low self‐efficacy [32, 33]. Among hospitalized patients with advanced HIV disease, mortality is higher among individuals who discontinued ART (29%) versus treatment‐naive patients (8%) [32]. Tuberculosis (TB), histoplasmosis and cryptococcosis are common causes of admission and deaths. TB is a leading cause of AIDS‐related deaths; 50% of estimated cases are identified, and treatment success is only 55%, resulting in 7800 annual deaths [34]. Only 29% of newly identified PWHIV receive TB prophylaxis, despite the availability of short‐course, affordable options [35]. Coformulated rifapentin‐isoniazid is helpful; in Brazil, coformulation increased treatment completion rates by 11% compared to isoniazid alone [36].

To reduce mortality, promoting the importance of early treatment and the principle of “undetectable = untransmittable” (U = U) is urgent [37]. In Brazil, Mexico and Peru, only 65% of PWHIV had an accurate picture of U = U [38]. Among HIV providers from Brazil and Mexico surveyed in 2020, 74% and 62% had an accurate understanding of this concept, potentially limiting U = U's ability to reduce stigma and motivate adherence [39].

HIV care remains largely centralized in specialized centres in most countries. Even when services are provided within primary care, they typically involve separate, specialized teams. There is a push to integrate HIV interventions across health systems, including via diagonal approaches [40], to scale‐up critical interventions for HIV elimination. This requires a strong primary care system including: adequate logistics, laboratories, information systems and motivated human resources delivering culturally competent, non‐discriminatory care. Innovative approaches to improved primary care have been piloted in the region: telemedicine [41, 42, 43], self‐testing [42, 44], home‐based interventions [35, 45], differential service delivery models and mhealth [46, 47, 48], but require scale‐up. Overall, these pilots reported high acceptability and feasibility in various service‐delivery settings, with stronger evidence for telemedicine, which maintained antiretroviral coverage and viral suppression. Effects on adherence and linkage‐to‐care were more mixed, with messaging improving adherence only in younger subgroups and high testing uptake inconsistently translating into treatment linkage.

Stigma is consistently identified by PWHIV and providers as what most undermines confidence and acceptance of HIV care decentralization [49]. Stigma is also particularly severe for cisgender women with HIV, who often experience an added layer of blame and moral judgement because dominant norms frame HIV as something they would not acquire [50, 51]. HIV‐related stigma and discrimination also impact those at risk of HIV. Data from Stigma Index 2.0 studies in Bolivia, Ecuador, Nicaragua, Paraguay and Peru showed that 15% of respondents faced discrimination in HIV‐related services and 27% in other health services [52]. Stigma data from the Caribbean remain limited, but the same survey in Jamaica found that 38% of respondents reported at least one experience of HIV‐related stigma or discrimination, while 47% feared verbal and 41% feared physical assault because of their HIV status [53]. In Brazil, 52.9% of respondents suffered some form of discrimination, 54.1% of transgender women and *travestis* did not seek healthcare due to anticipated transphobia and 21.4% of gay men were afraid avoided health services due to homophobia [54]. Transgender women and *travestis* face multiple and intersecting discrimination, often limiting their care engagement [55]. In some countries, restrictive legal frameworks and persistent discrimination against key populations create barriers to HIV testing, linkage and continuity of care. The criminalization of same sex relationships and the absence of protective laws for key populations [56] discourage diagnosis and limit uptake of prevention [57, 58].

### 1.3 HIV Prevention in LAC: A Focus on PrEP and PEP

HIV prevention remains an area for expansion in LAC [8, 59]. In 2024, an estimated 286,862 people used PrEP in the region, a level insufficient to generate public health impact [60]. Brazil started PrEP provision in December 2017, and accounts for the largest number of people using PrEP in the region, 165,473 (57.7%). Haiti and Mexico follow, each with approximately 30,000 PrEP users. There is, however, substantial regional interest in PrEP. A 2023 survey of key populations in nine Latin American countries showed that among HIV‐negative participants, only 19.3% were using PrEP, an additional 37.6% reported considering using PrEP if available, suggesting unmet need [61]. While national PrEP programmes or pilots have begun in English‐speaking Caribbean (Barbados, the Bahamas, Jamaica and the nine countries in the Organisation of Eastern Caribbean States), PrEP use is substantially higher in the Dominican Republic, Haiti and Cuba [60].

Meanwhile, the array of HIV prevention modalities is expanding. Alongside oral PrEP, a monthly dapivirine vaginal ring (DVR) and every 2‐month long‐acting cabotegravir (CAB‐LA) are recommended by WHO [62]. DVR could be useful especially in the Caribbean's more generalized epidemics but remains absent. CAB‐LA is not available programmatically in any LAC country, despite safety and efficacy data generated in clinical research sites from Argentina, Brazil and Peru [11]. Similarly, lenacapavir (LEN) remains unavailable across most of LAC, being recently approved in Brazil for HIV prevention [63, 64]. In Brazil, Unitaid‐funded studies are evaluating implementation of CAB‐LA and LEN to adults and adolescents [65, 66]. ImPrEP CAB‐Brasil demonstrated high acceptability and effectiveness of CAB‐LA, with 83% of participants choosing CAB over oral PrEP [67].

The PrEP implementation gap in LAC is multifactorial. In most countries, prescribing PrEP remains restricted to physicians, which limits the expansion of simplified delivery models like same‐day oral PrEP initiated by pharmacists, nurses and community PrEP programmes. Stigma and discrimination towards key populations, insufficient infrastructure, structural and social determinants further constrain PrEP uptake [68]. In Brazil, internalized homophobia and discrimination were associated with lower PrEP uptake and adherence and higher discontinuation [69, 70].

Improving PrEP coverage in LAC requires expansion of HIV testing, diversification of testing modalities and stronger use of network‐based strategies, in which peers or partners recruit from their social or sexual networks [71]. HIV self‐testing can play an important role in supporting this strategy through secondary distribution of kits by individuals initiating PrEP or undergoing testing. Evidence from Latin America shows high willingness to use HIV self‐testing among MSM and its potential to support linkage to PrEP [30]. Also, integrating sexually transmitted disease (STI) testing and treatment within PrEP services represents an important opportunity to expand prevention impact and address the high STI burden among key populations in LAC.

Modelling consistently shows the need to expand PrEP to impact population‐level HIV incidence. The PrEP Impact tool provides evidence on how PrEP coverage scenarios, product mixes and testing strategies could reduce new HIV acquisitions if implemented at scale. When implementing long‐acting options, increasing overall PrEP coverage should be a priority, rather than replacing existing users of oral PrEP with long‐acting formulations. Even with the introduction of long‐acting injectable PrEP, substantial epidemiological impact will only occur if a much larger proportion of those at elevated risk receive PrEP, underscoring the urgency of accelerating PrEP scale‐up.

Recent studies across various settings have identified choice as critical for both PrEP access and persistence, as they lead to increased coverage and more effective use [66, 72]. By integrating a combination of diverse and innovative delivery models, such as flexible clinic hours, PrEP dispensing machines in public transport stations, telehealth platforms and tailored health education, São Paulo, the epicentre of the Brazilian HIV epidemic, significantly expanded PrEP use (865% of registrations) and reduced new HIV diagnoses by 54% between 2016 and 2023 [73]. The ImPrEP project in Brazil, Mexico and Peru similarly underscored how PrEP can be embedded within public health programmes for same‐day PrEP initiation [74].

In 2024, WHO released updated guidelines recommending PEP access and distribution in community settings and task shifting towards demedicalization [75]. Yet, community delivery of PrEP remains rare, influenced by policy, regulatory and operational considerations to implementing prevention at scale, leading to missed opportunities for timely access [76].

## 2 Discussion

There have been important advances in the HIV response in LAC, but progress remains insufficient, with some countries, subnational areas and populations experiencing rising HIV incidence and a slower decline in AIDS‐related mortality than the global average. Structural and social determinants, including poverty, stigma, discrimination, gender inequalities, socioeconomic vulnerability, centralized service‐delivery and under‐resourced health systems, continue to limit timely diagnosis, continuity of treatment and uptake of prevention services [37, 39, 69, 70, 77, 78]. Laws and policies continue to adversely affect access for key populations [56], underscoring the need for context‐specific, rights‐based, community‐informed approaches [79].

Strengthening the integration of HIV services into primary healthcare across the region offers an important opportunity to overcome persistent gaps in access and continuity. Several Latin American countries, including Brazil, Uruguay, Cuba and Argentina, have advanced towards integrated service delivery models, with Paraguay and Peru implementing similar approaches [80]. However, care settings can be unwelcoming to key populations due to untrained staff or broader system limitations, including gender recognition, compromising service quality and trust. Innovative approaches for integration addressing these barriers are usually pilot studies that are expensive to scale up and/or sustain. This is particularly relevant within a global environment struggling with reduced funding for the HIV response. The region's heavy reliance on domestic funding has fostered autonomy, but also exposed it to fiscal fluctuations hindering scale‐up. Meanwhile, Latin America's contributions to clinical research and innovations demonstrate the strong potential for scale‐up. Unfortunately, restrictive patent agreements and regulatory hurdles delay advances reaching the populations most in need.

LAC faces a combination of shared and context‐specific challenges that require approaches capable of integrating lessons learned across countries while adapting to the cultural, social and structural diversity of the region. Achieving across‐region synergies will require stronger platforms for cross‐country learning, flexible service models that respond to local contexts and explicit political commitment to ensure the sustainability of prevention programmes, including stable financing and long‐term partnerships with communities [79]. Together, these elements create conditions for a more resilient and equitable HIV response capable of advancing towards elimination goals in LAC.

## 3 Conclusions

Persistent gaps in early HIV prevention and care continuum indicate that current health system capacities and investments remain insufficient to achieve population‐level impact in LAC. Strengthening integration with primary healthcare, ensuring community participation and addressing structural determinants such as stigma and discrimination are essential to improve continuity along the HIV cascade. The region's longstanding community leadership and scientific innovation offer critical opportunities to expand evidence‐based prevention, including differentiated PrEP and PEP delivery, community‐led testing and self‐testing. In the context of rising chronic disease burdens, sustained domestic investment in health systems is required to modernize service delivery and support long‐term adherence and viral suppression. Ensuring sustainability, particularly as the global health funding landscape is being reconfigured, will require political commitment, efficient resource allocation and stronger partnerships with civil society.

## Author Contributions

MP and KAK conceived the manuscript and led the drafting of the initial version. OS, HP, AB, TST, MA, VV and BG provided critical input and reviewed successive drafts. All authors reviewed and approved the final submitted version.

## Conflicts of Interest

All authors declare no conflicts of interest.

## Data Availability

These data were derived from the following resources available in the public domain: https://www.paho.org/en/hiv‐situation‐americas. Additional data from UNAIDS were used; these data are available upon request.
